# Corrosion Behavior of 3104 Aluminum Cans When Used as Packaging for Chinese Liquor

**DOI:** 10.3390/ma17163884

**Published:** 2024-08-06

**Authors:** Mingjie Fan, Jinyang Chen, Jie Gu, Zheying Wu

**Affiliations:** 1 Chemical Engineering and Technology, Shanghai University, Shanghai 200444, China; 2 Shanghai Bao Steel Packaging Co., Ltd., Shanghai 200949, China

**Keywords:** aluminum cans, Chinese liquor, corrosion, migration, EIS

## Abstract

Aluminum cans are commonly used for packaging soft drinks and low-alcohol beverages due to their good recyclability. To enhance the economic cycle and expand the packaging of liquors, the feasibility of commercial 3104 aluminum cans for packaging Chinese liquor was studied. The aluminum’s migration into alcoholic solutions was studied using inductively coupled plasma emission spectroscopy (ICP-OES). Electrochemical impedance spectroscopy (EIS) was used to study the corrosion process of epoxy coatings on the aluminum cans. Scanning electron microscopy (SEM), energy dispersive X-ray spectroscopy (EDS), infrared attenuated total reflection (IR-ATR), and X-ray diffraction (XRD) were used to determine the inner coatings and adhering surfaces of the cans and the corrosion process. The results showed that the maximum aluminum migration in Chinese liquor was 4.3572 mg/kg at 60 °C for 30 days. The epoxy coating was corroded enough to decrease the coating impedance and expose the metal substrate after 25 days. Permeation and aging degradation of coatings are the main factors to consider when packaging liquor.

## 1. Introduction

Aluminum is widely used for food packaging because of its low density, high mechanical strength, high recycling rate, and corrosion resistance. To date, metal cans have become a popular form of packaging around the world and more than 250 billion cans are used every year [1], and aluminum cans have gradually become the main type of metal beverage cans. At present, aluminum cans are mainly used to package water-based soft drinks and low-alcohol beverages such as carbonated drinks and beer. It is known that aluminum alloys exhibit corrosion in anhydrous ethanol [2,3] and its water solutions [4,5,6,7]. Similarly, the original corrosion resistance of aluminum cans may be altered when they are in contact with liquor for long time and exposed to the combining effects of multiple complex components [8]. However, there are few studies about the use of aluminum cans for the packaging of liquors.

In order to reduce the corrosion of metal in contact with beverages, commercial aluminum cans are coated with a polymer coating as a food contact material (FCM) [9]. These anti-corrosive coatings are complex mixtures that consist of vinyl or phenolic lacquers and epoxy resins [10]. Epoxy resins are very important because of their heat resistance, mechanical strength, and corrosion resistance [11]. As a thermosetting polymer, epoxy resins can form strong chemical bonds on the surface of metal cans. Although cans have this coating as a surface barrier, corrosion sometimes still occurs as a result of beverages. The liquid diffuses into the polymer layer and affects the packaging barrier [12]. After this liquid permeation occurs and the coating swells, corrosion and leakage of the aluminum can occur, and excessive aluminum migrating into beverages can cause health hazards such as Alzheimer’s disease, Parkinson’s syndrome, and multiple sclerosis [13].

The corrosion processes of metal-coated cans are usually estimated using electrochemical techniques [14], and electrochemical impedance spectroscopy (EIS) is a relatively fast and reliable technique for the evaluation of protective coatings. The corrosion resistance of the inner surface of aluminum cans for guarana, cola-flavored, and tonic water soft drinks were evaluated with EIS by Esteves et al. [15]. The degradation of aluminum can coatings for Green Cola and Red Bull cans was examined by Almoiqli et al. [16].

The main purpose of this study was to determine the feasibility of 3104 aluminum alloy cans for packaging Chinese liquor and to study the related corrosion processes. Inductively coupled plasma emission spectroscopy (ICP-OES) and EIS were used to determine the leakage of aluminum and the corrosion of the cans. In addition, scanning electron microscopy and X-ray microanalysis (SEM-EDS), infrared attenuated total reflection (IR-ATR), and X-ray diffraction spectroscopy (XRD) were employed to measure the internal coatings and the adhering surfaces of the cans. The corrosion mechanism of the liquor was determined.

## 2. Materials and Methods

### 2.1. Samples and Reagents

3104 aluminum alloy-coated cans were supplied by Shanghai Baosteel Packaging Co., Ltd. (Shanghai, China), and 52% vol. strong-flavored Chinese liquor was purchased from Harbin Junmin Liquor Co., Ltd. (Harbin, Heilongjiang Province, China). Nitric acid (GR, 65–68%) was obtained from Shanghai Sinopharm Chemical Reagent Co., Ltd. (Shanghai, China). Anhydrous ethanol (AR, 99.7%) was obtained from Shanghai Boer Chemical Reagent Co., Ltd. (Shanghai, China). Al standard solution (1000 mg/L) was acquired from Tanmo Quality Inspection Technology Co., Ltd. (Beijing, China).

### 2.2. Migration Test of Aluminum Cans

According to EU No. 10/2011 [17] and GB 31604.1-2015 [18] criteria, 20% ethanol, 50% ethanol, 95% ethanol, and 52% Chinese liquor were selected as the food-packaging simulants. They were poured into the aluminum-coated cans, and the cans were sealed and then put into a thermostatic oven at 60 °C. Test times were set at 5, 10, 15, 20, 25, and 30 days. The condition of 10 days at 60 °C corresponds to long-term storage above 6 months at room temperature and below [17,18]. Based on the appropriate extension of this specific migration condition, the condition of 30 days at 60 °C is about equivalent to a simulation of 18 months of shelf life at room temperature.

After migration, the solutions were evaporated to dryness on a thermostatic electrothermal plate, subsequently dissolved and fixed with 2% aqueous nitric acid solution, and then filtered for ICP-OES analysis. Aluminum cans soaked for different lengths of time were used for the subsequent characterization and electrochemical impedance spectroscopy.

Aluminum migration was calculated as M = (C_t_ − C_0_) × V/m, where M is the amount of aluminum migration in the medium (μg/kg); C_t_ and C_0_ are the concentrations of the migration and blank samples (μg/L); V is the volume of the migration sample (mL); and m is the mass of the sample of the migration solution before digestion (g).

### 2.3. Characterization and Analysis

The Al concentration was analyzed with ICP-OES (Optima 8000, PerkinElmer, Waltham, MA, USA). Electrochemical impedance spectroscopy (EIS) of the soaked aluminum can was measured using an electrochemical analysis workstation (CHI604E, CH Instruments, Shanghai, China). The morphology of the aluminum cans during corrosion was examined with scanning electron microscopy (SU1510, Hitachi, Tokyo, Japan). IR-ATR (Nicolet 380, ThermoFisher, Waltham, MA, USA) was used to study the changes in the properties of the epoxy coating. X-ray diffraction (D2 Phaser, Bruker, Billerica, MA, USA) was used to determine the changes in the corrosion composition of the cans.

The operation parameters of the ICP-OES were as follows: RF power: 1250 W; plasma gas flow rate: 15.0 L/min; auxiliary gas flow rate: 0.9 L/min; nebulizer gas flow rate: 0.85 L/min; sample flow rate: 1.5 mL/min; and Al wavelength: 396.153 nm.

EIS measurements were performed to obtain a stable open-circuit potential by applying a sinusoidal voltage of ±20 mV in a frequency range of 10^−2^ to 10^5^. A three-electrode cell system was constructed at room temperature, containing a saturated calomel electrode as a reference electrode, a platinum electrode as a counter electrode, and the aluminum can as the working electrode.

## 3. Results and Discussion

### 3.1. Aluminum Migration

Inductively coupled plasma optical emission spectroscopy (ICP-OES) can be used to determine the concentration of elemental components in samples, and it was used in this study to determine the migration of aluminum. The results are given in Table 1 and show that the migration of aluminum increased with longer soaking time. As for the three different aqueous ethanol solutions, the maximum migration of aluminum was observed in the 50% ethanol, followed by the 20% ethanol, and the least migration of aluminum was observed in the 95% ethanol. However, the migration of aluminum in the Chinese liquor was much higher than that observed in the other simulants. This result may be attributed to the complex composition of various trace compounds contained in Chinese liquor [19].

**Figure 1:**
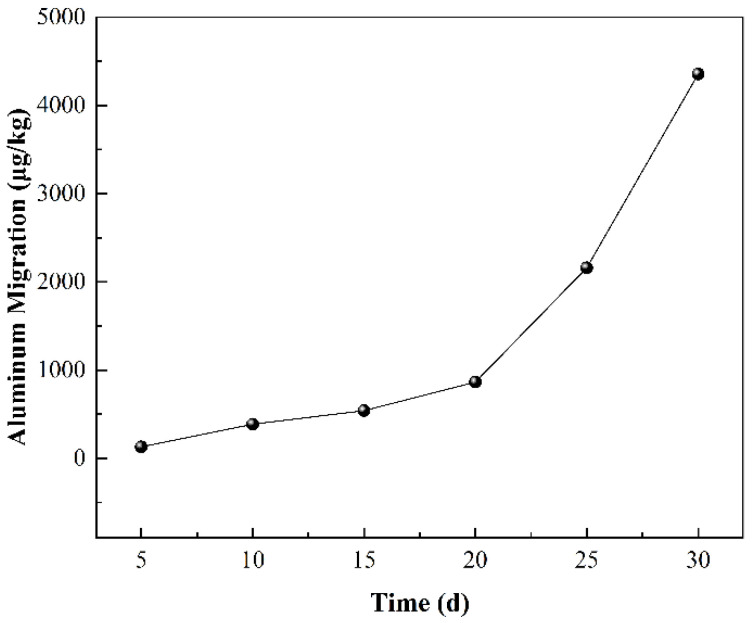
The aluminum migration in 52% Chinese liquor at 60 °C at different times.

To further understand the interaction between cans and Chinese liquor, the migration of aluminum in the actual liquor was studied across more time points (Figure 1). The results show that the migrated amount of aluminum increased with time. The maximum migrated amount of aluminum in the Chinese liquor was about 4.3572 mg/kg, which is equivalent to an aluminum concentration of about 4 mg/L in the migration sample. This is much higher than the accepted minimum contamination level of 0.2 mg/L concentration outlined by the American Agency for Toxic Substances and Disease Registry (ATSDR) [20]. This indicates that Chinese liquor stored in aluminum cans has serious health risks.

### 3.2. Corrosion Morphology of Aluminum Cans

**Figure 2:**
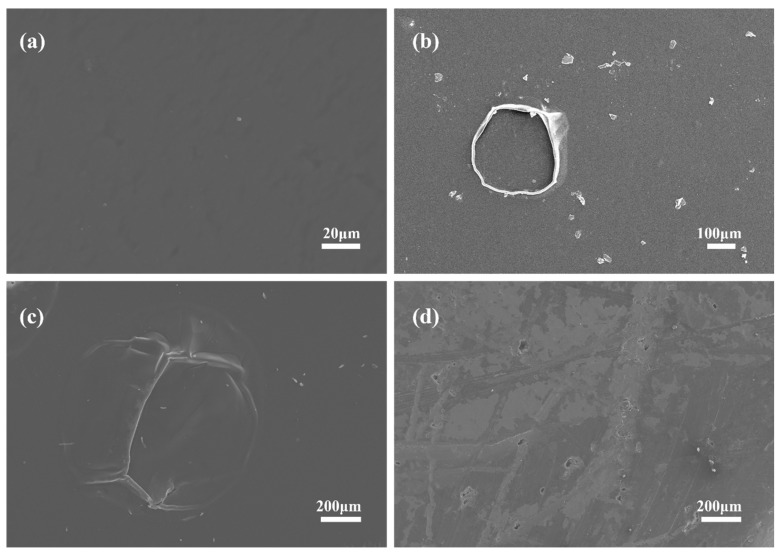
SEM images of the internal surfaces of aluminum cans after different durations of soaking: (**a**) 0, (**b**) 5, (**c**) 10, and (**d**) 30 days in Chinese liquor.

Figure 2 shows the surface morphology of the coated sample cans after being soaked in Chinese liquor for different lengths of time, as observed by SEM. The surface coating of the unsoaked cans was relatively flat, whereas after 5 days, circular bubble precursors emerged between the coating and the surface of the aluminum alloy without apparent cracking of the coating. After soaking for more than 10 days, the surface of the coated cans became rougher with localized bulging and cracking. After 30 days of soaking, a corroded area was clearly visible on the surface of the sample. The coating lost its basic protection, and the aluminum alloy substrate developed widespread exposure and numerous small pits.

Table 2 shows the EDS analysis of the sample cans after soaking for 10 and 30 days in Chinese liquor. The content of the can soaked for 10 days was Al = 9.0, C = 81.5, O = 9.6, and the presence of the epoxy coating resulted in a higher percentage of carbon content. After 30 days of corrosion, the aluminum content drastically increased due to the damage of the coating (Al = 38.1, C = 19.5, O = 42.4), and the increase in the oxygen content indicates the formation of corrosive substances.

**Figure 3:**
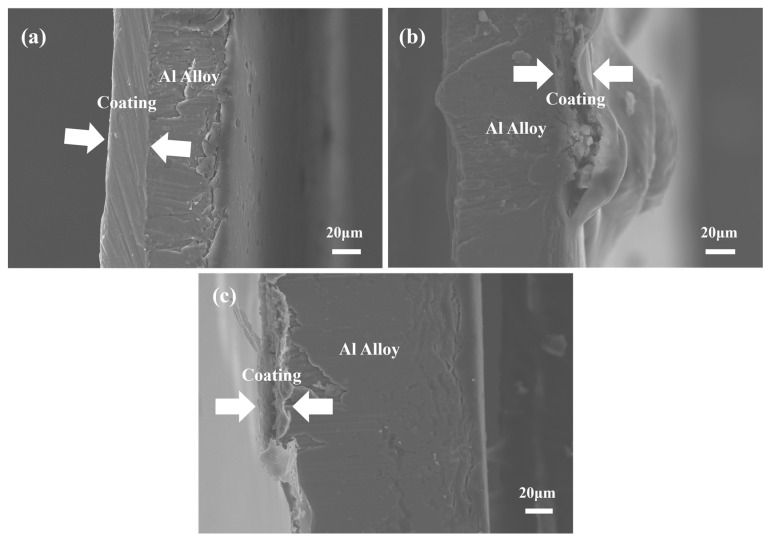
Cross-sectional morphologies of aluminum-coated cans soaked for (**a**) 0, (**b**) 10, and (**c**) 30 days in Chinese liquor.

Furthermore, the cross-sectional structure of the aluminum cans during the corrosion process with Chinese liquor is given in the SEM images below (Figure 3). It can be observed that the thickness of the can was about 100 μm and the layer depth of the epoxy coating (FCM) was about 20 μm. The arrows show the states of the epoxy coating. At first, the inter-layer connection between the coating and the metal substrate was tight. After 10 days of soaking, a gap existed between the coating and the alloy substrate, and there were granular corrosion products encapsulated inside the bulging position. In this case, corrosion happened through infiltration, and the volumetric expansion stress of the corrosion products accelerated the damage of the coating. After 30 days, the cross-section became coarser and the inter-layer gap was obvious. The coating fell off in the form of small debris. Thus, the coating was no longer integral in covering the substrate.

### 3.3. Change in Epoxy Coating

**Figure 4:**
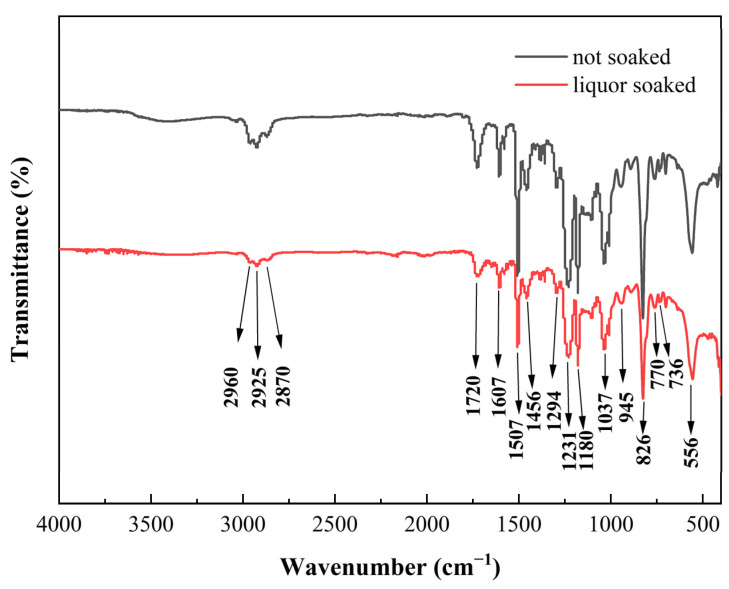
The IR-ATR spectra of epoxy coating before and after soaking for 30 days.

Figure 4 gives the IR spectra of the epoxy resin before and after soaking for 30 days in Chinese liquor. The band at 2925 cm^−1^ is attributed to the stretching vibration of the aliphatic methylene (-CH_2_-), and the low-intensity peaks located at 2960 and 2870 cm^−1^ indicate the stretching modes of aliphatic methyl groups (-CH_3_). The peak at 1720 cm^−1^ is the stretching vibration peak of the carbonyl groups (C=O). The stronger absorption bands at 1607, 1507, and 1456 cm^−1^ are the vibrations of the aromatic benzene ring, and the bending of the hydroxyl groups is seen at 1294 cm^−1^. The stretching vibration peaks of the aromatic ether are at 1231 and 1180 cm^−1^, while the stretching mode of alkyl ether is at 1037 cm^−1^. The stretching of the epoxy groups appears at 945 and 770 cm^−1^ [21]. C-H aromatic bending patterns can be noticed at 826, 736, and 556 cm^−1^.

Comparing the infrared spectra before and after soaking, it was found that the intensity of the vibrational absorption peaks of the aromatic benzene ring at 1507 cm^−1^ was significantly weakened; the intensity of the aromatic ether and the alkyl ether stretching peaks at 1231 and 1180 cm^−1^, as well as that of the alkyl ether stretching absorption peak at 1037 cm^−1^, was reduced; and there was an attenuation of the C-H aromatic bending absorption at 826 cm^−1^. This indicates that soaking with liquor has a disruptive impact on the polymeric structure of epoxy resin, and leads to degradation.

### 3.4. Change in Composition of Aluminum Cans

**Figure 5:**
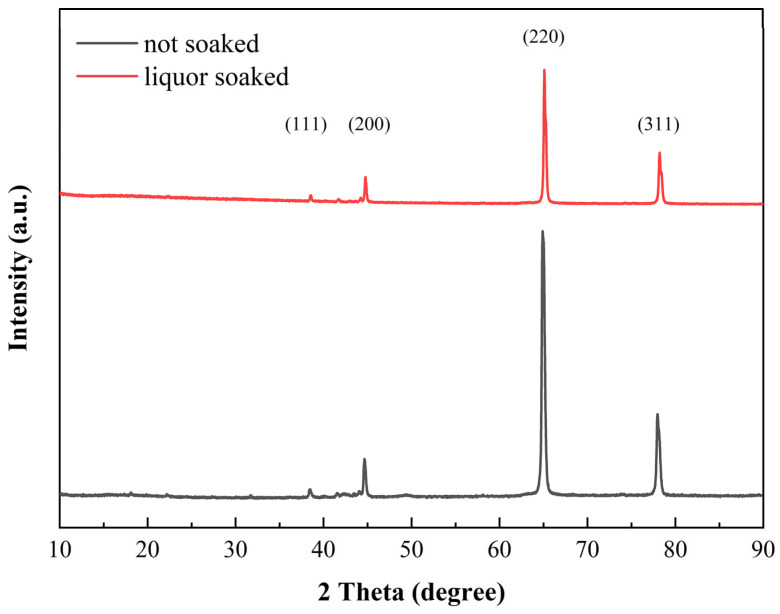
The XRD patterns of the aluminum cans before and after corrosion for 30 days.

Figure 5 shows the XRD patterns of the aluminum cans before and after soaking for 30 days in Chinese liquor. There was no significant change between the locations of all of the strong feature peaks prior to corrosion and their locations after corrosion. The peak signals with 2-theta angles of 38.5, 45.5, 65.2, and 78.2° were attributed to metallic pure aluminum (JCPDS No. 89-4037). For the above-indexed signal patterns, there were three different states of aluminum phases: diffraction peaks with 2-theta angles of 38.5 and 65.2° were attributed to boehmite (AlOOH); those with 2-theta angles of 65.2 and 78.2° were identified as face-centered cubic aluminum; and that with a two-theta angle of 45.5° belonged to cubic γ-Al_2_O_3_ [22]. Moreover, there were some slight diffraction signals between the positions of the (111) and (200) crystal planes, which were related to the effects of impurities such as manganese, magnesium, iron, and copper in the 3104 aluminum alloy [23]. The intensity of the (220) and (311) crystal plane peaks of the soaked samples experienced a dramatic decline compared to those of the unsoaked aluminum cans. This was due to corrosion during the soaking process.

### 3.5. Electrochemical Impedance Spectroscopy

**Figure 6:**
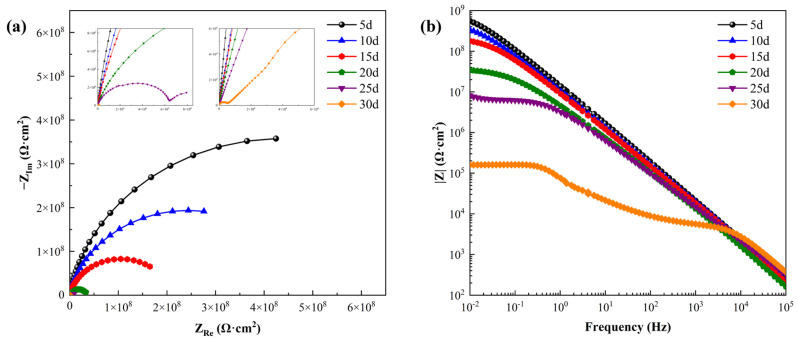
EIS plots of aluminum-coated cans soaked in Chinese liquor: (**a**) Nyquist plot; (**b**) Bode plot.

EIS was used to determine the anti-corrosion process of epoxy resin coatings treated with Chinese liquor and soaked at 60 °C for 30 days. Figure 6 shows the Nyquist and Bode impedance spectra of 3104 aluminum alloy cans soaked in Chinese liquor for 30 days. Within the first 5 days of immersion, the Nyquist plot shows an incomplete depressed semicircular arc with a large diameter and the Bode plot exhibits a curve with a slope close to −1, and the low-frequency modulus was recorded to be 5.55 × 10^8^ Ω·cm^2^. At this time, the organic coating was relatively intact and could be considered as an isolation layer with a large resistance value and a small capacitance value.

As the soaking time increases, the overall diameter of the capacitive arc of the Nyquist plot decreases continuously, and the low-frequency impedance modulus value of the corresponding Bode plot also continues to decrease. The curves of each Bode plot nearly overlap each other in the high-frequency range, transiting to a relatively smooth plateau in the low-frequency range.

It is significant that a straight line superimposed at 45° to both axes following the semicircle of the capacitance arc appeared in the Nyquist plot after soaking for 25 days, which corresponded to the process of substance diffusion [24]. The appearance of a diffusion tail at low frequencies means that the coating was broken, and thus the liquor easily contacted the surface of the aluminum alloy, and the electrode process was dominated by the metal substrate [25]. When the can was soaked in liquor for 30 days, the low-frequency modulus in the Bode plot dropped to a minimum of 1.60 × 10^5^ Ω·cm^2^, indicating that the coating lost its anti-corrosion effect, which corresponded to the macroscopic appearance of partial pitting corrosion.

The bubbling of the coating during the initial 20 days suggests two time-constant characteristics. Nevertheless, the Nyquist plots kept a larger depressed semicircular arc, without a clear two-segment capacitance arc for the two time constants. This can be ascribed to the overlapping of the small and large capacitive arcs, making it difficult to distinguish the two time constants [26]. One of the capacitive impedance arcs in the high-frequency range reflects the property of the epoxy coating, while the capacitive impedance arc in the low-frequency range may be related to the corrosion reaction on the surface of the aluminum alloy.

**Figure 7:**
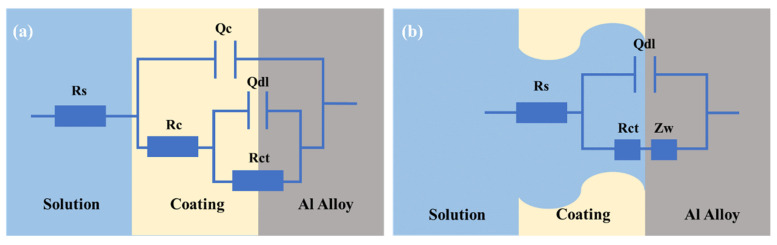
Equivalent circuits proposed for aluminum-coated cans during different phases of soaking: (**a**) phase one; (**b**) phase two.

After 25 days of soaking, the impedance plots began to exhibit a combination of the capacitive arc and diffusion tail. The capacitive arc at higher frequencies and the diffusion impedance tail at lower frequencies can be attributed to the charge transfer process of the metal substrate and the diffusion process of the corrosion substances, respectively [27]. The electrochemical impedance curves for two time phases can be illustrated by the equivalent circuits in Figure 7 [28].

**Figure 8:**
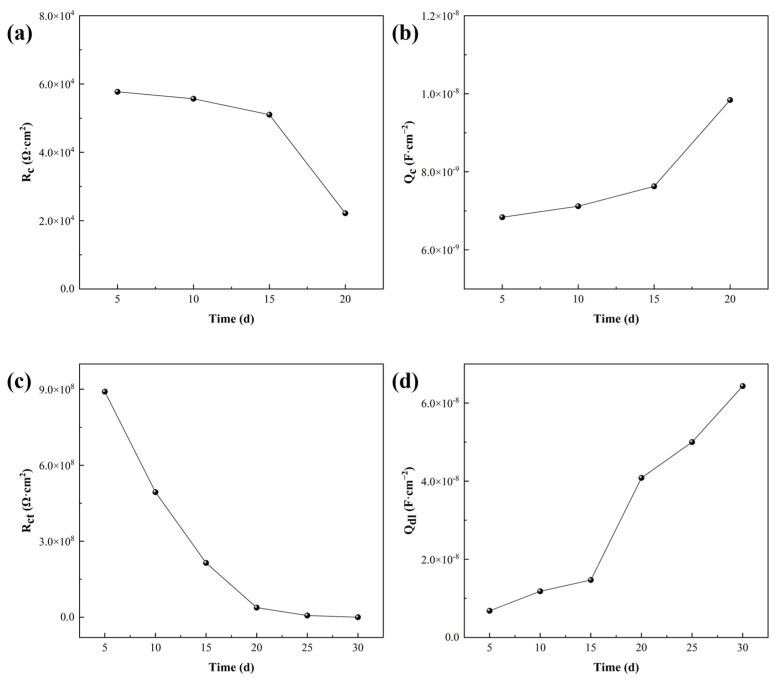
Changes in the fitted parameters with soaking time: (**a**) coating resistance R_c_ curve; (**b**) coating capacitance Q_c_ curve; (**c**) charge transfer resistance R_ct_ curve; (**d**) electric double-layer capacitance Q_dl_ curve.

For a quantitative evaluation of the coating performance, the determined electrochemical impedance spectra were further analyzed by using the established equivalent physical models. The values of the electrochemical parameters of the fitted equivalent components are shown in Table 3. These circuit elements are interpreted as the solution resistance (R_s_), coating interface capacitance (Q_c_), electric double-layer capacitance (Q_dl_), resistance of the conductive path through the coating (R_c_), charge transfer resistance (R_ct_), and diffusion (Warburg) impedance (Z_w_), respectively. Figure 8 shows the change in each essential parameter related to the coating/substrate with the soaking time. For 30 days in liquor, the electrical resistance of the epoxy coating continued to decrease, while its interfacial capacitance continued to increase. It is indicated that the liquor continuously infiltrated into the coating through the micropores caused by the manufacturing process, and the enlarged porosity gradually weakened the anti-corrosion effect. The decrease in charge transfer resistance and the rise in double-layer capacitance may be related to the regional exposure of the aluminum alloy substrate and the further corrosion of the oxide layer on the aluminum surface.

According to the EIS analysis and the characterization of the morphology, it is known that the coating became damaged as a result of both the degradation of the coating itself and the destruction of the corrosion products due to stress expansion under the coating.

## 4. Conclusions

Here, the feasibility of packaging Chinese liquor with 3104 aluminum-coated cans was studied. The results show that food simulants cannot completely replace liquor in a packaging test. The level of alcohol alone is not an important factor in the packaging of liquor with aluminum cans. The epoxy coating on the aluminum cans could not resist the corrosion caused by the Chinese liquor. The degradation of the coating and the corrosion of the aluminum, as determined using IR and XRD, and the surface and cross-sectional morphologies showed damage to the coating caused by the liquor. Electrochemical impedance spectroscopy can be used to analyze the corrosion of aluminum-coated cans. As for the liquor, the degradation of the coating resulted in an excessive migration of Al into the packaged liquor.

## Figures and Tables

**Table 1:** Aluminum migration (μg/kg) under different conditions.

Time (d)	Aluminum Migration (μg/kg)			
	Ethanol 20% (*v*/*v*)	Ethanol 50% (*v*/*v*)	Ethanol 95% (*v*/*v*)	Chinese Liquor 52% (*v*/*v*)
10	12.16	17.76	2.56	387.39
30	13.89	24.02	7.07	4357.2

**Table 2:** EDS analysis of aluminum-coated cans after soaking for 10 and 30 days in Chinese liquor.

Time (d)	Element (At%)			
	Carbon	Oxygen	Aluminum	Total
10	81.5	9.6	9.0	100.0
30	19.5	42.4	38.1	100.0

**Table 3:** Parameter values of electrochemical components at different soaking times.

Time (d)	5	10	15	20	25	30
R_c_ (Ω·cm^2^)	5.773 × 10^4^	5.568 × 10^4^	5.103 × 10^4^	2.217 × 10^4^	-	-
Q_c_ (F·cm^−2^)	6.837 × 10^−9^	7.119 × 10^−9^	7.629 × 10^−9^	9.830 × 10^−9^	-	-
R_ct_ (Ω·cm^2^)	8.906 × 10^8^	4.940 × 10^8^	2.143 × 10^8^	3.793 × 10^7^	6.554 × 10^6^	6.450 × 10^3^
Q_dl_ (F·cm^−2^)	6.828 × 10^−9^	1.182 × 10^−8^	1.470 × 10^−8^	4.084 × 10^−8^	5.000 × 10^−8^	6.433 × 10^−8^

## Data Availability

Data are unavailable due to privacy and ethical restrictions.

## References

[1] Kacmary P., Rosova A., Sofranko M., Bindzar P., Saderova J., Kovac J. (2021). Creation of Annual Order Forecast for the Production of Beverage Cans—The Case Study. Sustainability.

[2] Calabrese L., Bruzzaniti P., Proverbio E. (2018). Pitting corrosion of aluminum alloys in anhydrous ethanol. Mater. Corros..

[3] Gazenbiller E., Arya V., Reitz R., Engler T., Oechsner M., Höche D. (2021). Statistical analysis of AA-1050 localized corrosion in anhydrous ethanol. Corros. Sci..

[4] Brito-Franco A., Uruchurtu J., Rosales-Cadena I., Lopez-Sesenes R., Serna-Barquera S.A., Hernandez-Perez J.A., Rocabruno-Valdes C., Gonzalez-Rodriguez J.G. (2020). Corrosion Behavior of Al in Ethanol–Gasoline Blends. Energies.

[5] Park I.J., Yoo Y.H., Kim J.G., Kwak D.H., Ji W.S. (2011). Corrosion characteristics of aluminum alloy in bio-ethanol blended gasoline fuel: Part 2. The effects of dissolved oxygen in the fuel. Fuel.

[6] Park I.-J., Nam T.-H., Kim J.-H., Kim J.-G. (2014). Evaluation of corrosion characteristics of aluminum alloys in the bio-ethanol gasoline blended fuel by 2-electrode electrochemical impedance spectroscopy. Fuel.

[7] Song G.-L., Liu M. (2013). Corrosion and electrochemical evaluation of an Al–Si–Cu aluminum alloy in ethanol solutions. Corros. Sci..

[8] Jellesen M.S., Rasmussen A.A., Hilbert L.R. (2006). A review of metal release in the food industry. Mater. Corros..

[9] Kato L.S., Conte-Junior C.A. (2021). Safety of Plastic Food Packaging: The Challenges about Non-Intentionally Added Substances (NIAS) Discovery, Identification and Risk Assessment. Polymers.

[10] Lestido-Cardama A., Vazquez Loureiro P., Sendon R., Paseiro Losada P., Rodriguez Bernaldo de Quiros A. (2021). Application of chromatographic analysis for detecting components from polymeric can coatings and further determination in beverage samples. J. Chromatogr. A.

[11] Blanco I., Cicala G., Costa M., Recca A. (2006). Development of an epoxy system characterized by low water absorption and high thermomechanical performances. J. Appl. Polym. Sci..

[12] Soares D.S., Bolgar G., Dantas S.T., Augusto P.E.D., Soares B.M.C. (2019). Interaction between aluminium cans and beverages: Influence of catalytic ions, alloy and coating in the corrosion process. Food Packag. Shelf Life.

[13] Brylinski L., Kostelecka K., Wolinski F., Duda P., Gora J., Granat M., Flieger J., Teresinski G., Buszewicz G., Sitarz R. (2023). Aluminium in the Human Brain: Routes of Penetration, Toxicity, and Resulting Complications. Int. J. Mol. Sci..

[14] Wang Y.-C., Su M., Xia D.-H., Wu Z., Qin Z., Xu L., Fan H.-Q., Hu W. (2019). Development of an electrochemical sensor and measuring the shelf life of tinplate cans. Measurement.

[15] Esteves L., Garcia E.M., Castro M.d.M.R., Lins V.F.C. (2014). Electrochemical study of corrosion in aluminium cans in contact with soft drinks. Corros. Eng. Sci. Technol..

[16] Almoiqli M., Alharbi K.N., Alnuwaiser M.A., Yajizi G., Alshoshan S., Baduways W., Albeladi M.I., Alsanea R.S., Aljohani T.A. (2023). Corrosion Behavior of Aluminium-Coated Cans. Materials.

[17] European Commission Commission Regulation (EU) No 10/2011 of 14 January 2011 on Plastic Materials and Articles Intended to Come into Contact with Food Text with EEA Relevance. 2011. pp. 1–89. https://eur-lex.europa.eu/legal-content/EN/TXT/?uri=CELEX%3A32011R0010&qid=1722908422783.

[18] (2015). National Standards for Food Safety General Rules for Migration Testing of Food Contact Materials and Products.

[19] Du P., Jiao G., Zhang Z., Wang J., Li P., Dong J., Wang R. (2023). Relationship between Representative Trace Components and Health Functions of Chinese Baijiu: A Review. Fermentation.

[20] ATSDR (2008). Pubilc Health Statement Aluminum CAS # 7429-90-5 Division of Toxicology and Environmental Medicine. www.atsdr.cdc.gov.

[21] Liu X., Shao Y., Zhang Y., Meng G., Zhang T., Wang F. (2015). Using high-temperature mechanochemistry treatment to modify iron oxide and improve the corrosion performance of epoxy coating—I. High-temperature ball milling treatment. Corros. Sci..

[22] Nnaji N., Nwaji N., Mack J., Nyokong T. (2019). Corrosion Resistance of Aluminum against Acid Activation: Impact of Benzothiazole-Substituted Gallium Phthalocyanine. Molecules.

[23] López-Juárez R., Razo-Perez N., Pérez-Juache T., Hernandez-Cristobal O., Reyes-López S.Y. (2018). Synthesis of α-Al2O3 from aluminum cans by wet-chemical methods. Results Phys..

[24] Huang X., Li N. (2007). Structural characterization and properties of lanthanum film as chromate replacement for tinplate. Appl. Surf. Sci..

[25] Zhou C., Wang J., Song S., Xia D., Wang K., Shen C., Luo B., Shi J. (2013). Degradation mechanism of lacquered tinplate in energy drink by in-situ EIS and EN. J. Wuhan Univ. Technol.-Mater. Sci. Ed..

[26] Chu Z., Deng W., Zheng X., Zhou Y., Zhang C., Xu J., Gao L. (2020). Corrosion Mechanism of Plasma-Sprayed Fe-Based Amorphous Coatings with High Corrosion Resistance. J. Therm. Spray Technol..

[27] Macák J., Sajdl P., Kučera P., Novotný R., Vošta J. (2006). In situ electrochemical impedance and noise measurements of corroding stainless steel in high temperature water. Electrochim. Acta.

[28] Bonora P.L., Deflorian F., Fedrizzi L. (1996). Electrochemical impedance spectroscopy as a tool for investigating underpaint corrosion. Electrochim. Acta.

